# Exercise Therapy Teamwork in German Rehabilitation Settings: Results of a National Survey Using Mixed Methods Design

**DOI:** 10.3390/ijerph18030949

**Published:** 2021-01-22

**Authors:** Judith Wais, Wolfgang Geidl, Nina Rohrbach, Gorden Sudeck, Klaus Pfeifer

**Affiliations:** 1 Institute of Sport Science, Eberhard Karls University of Tübingen, 72074 Tübingen, Germany; judith.wais@uni-tuebingen.de (J.W.); gorden.sudeck@uni-tuebingen.de (G.S.); 2 Department of Sport Science and Sport, Friedrich-Alexander University Erlangen-Nürnberg, 91058 Erlangen, Germany; klaus.pfeifer@fau.de; 3 Department of Sport and Health Sciences, Technical University of Munich, 80809 Munich, Germany; nina.rohrbach@tum.de

**Keywords:** interprofessional practice, teamwork, physical therapy, physical activity promotion, survey, mixed methods

## Abstract

A key prerequisite for implementing biopsychosocial exercise therapy concepts as parts of multimodal rehabilitation programs is interprofessional teamwork. Based on a nationwide survey of exercise therapy using a mixed methods design, it is of interest to determine to what extent there are links between team-related processes (e.g., interprofessional exchange) and structural features of the exercise therapy departments (e.g., department size) and the individual rating of interprofessional teamwork. The first part of the study involved a questionnaire-based survey, where exercise therapy heads of 1146 rehabilitation facilities were contacted. In the second part of the study, 58 exercise therapy heads held discussions in six focus groups. The results from both parts showed that interprofessional teamwork was rated positively overall. Team meetings were seen as the central platform for exchange. However, particularly in larger facilities, the hierarchical position of medical management and lacking resources were negatively associated with interprofessional exchange. The results affirm empirically that a more binding provision of adequate structural and organizational conditions, such as sufficient time slots for liaising on content, are essential for effective teamwork. This would facilitate and improve the promotion of physical activity in multimodal rehabilitation programs.

## 1. Introduction

Positive proof of the effectiveness of exercise therapy now exists for a large number of diseases, such as [1]. Consequently, exercise therapy plays a major role in multimodal rehabilitation programs and accounts for about 35% of therapeutic services within inpatient, as well as outpatient rehabilitation programs in German rehabilitation settings [2]. According to quality guidelines of the most important cost providers of these rehabilitation programs in Germany, exercise therapy has to be embedded into biopsychosocial rehabilitation concepts [3], which is in line with international discussions on refinement of exercise therapy towards elaborated biopsychosocial therapy concepts, such as [4]. This means that traditional concepts focusing on physical training alone had to be expanded in terms of psychosocial and behavioral goals in order to achieve long-term changes of physical activity behavior and ensure sustainability of health benefits. A key prerequisite for implementing such biopsychosocial concepts as part of rehabilitation programs is interprofessional teamwork [5].

Interprofessional teamwork is characterized by different professions with differing skills and functions working together collaboratively in a team in order to ensure the common task of holistic treatment planning and therapy implementation that is tailored to the rehabilitation patients. Interprofessional teamwork is seen as a central quality feature in multimodal rehabilitation [6] and is gaining in significance, also on account of the advancing specializations and distinctions between specialist disciplines, as these can lead to a loss of the holistic approach on the part of the individual disciplines [7]. Nevertheless, the nature and scope of interprofessional teamwork in Germany varies considerably [5].

Effective teamwork has a positive influence, both at the level of the rehabilitation patients (e.g., better treatment outcomes like patients’ functional status or health-related quality of life as well patient satisfaction) [8] and at the level of the team members (e.g., increased job satisfaction) [5]. The nature of the teamwork hinges on two main themes: team processes and organizational team structure [7]. From a joint consideration of six international reviews [5,9,10,11,12,13], Müller et al. [6] identified a selection of 10 process-based (e.g., sufficient flow of information and open communication in the team) and six organizational (e.g., appropriate financial and administrative resources and appropriate team size) determinants of successful interprofessional teamwork.

It was also shown that organizational conditions had an influence on teamwork in multimodal rehabilitation in Germany. For example, there were different prerequisites for acting interprofessionally depending on the team size or the health condition in question, such as [14]. In addition, in terms of the rehabilitative healthcare system in Germany, the setting of the rehabilitation as an in-patient or an out-patient measure is a possible factor that influences teamwork.

The majority of the research done on teamwork refers to the rehabilitation team as a whole, as interprofessional teamwork is seen as a basis for the implementation of biopsychosocial therapy concepts, such as [14]. The team members generally stem from the professional areas of psychology, nursing care, social work/social care, sports science/exercise therapy, physiotherapy, ergotherapy, ecotrophology, and the medical profession [15]. Alongside interprofessional teams, in multimodal rehabilitation there are also intraprofessional teams, such as nursing care teams at a medical station. This means that the same persons are often members of different teams at the same time (“multiple team membership” [16]) and collaboration takes place both at an interprofessional and an intraprofessional level. In this regard, exercise therapy practitioners are also generally members of an interprofessional rehabilitation team as a whole, as well as members of an intradisciplinary exercise therapy team (physiotherapists and sports therapists/sports scientists). As a result, research on exercise interventions in rehabilitation settings should focus both on the collaboration within a team and between different teams [17].

However, there are scarcely any findings to date regarding how collaboration in exercise therapy teams in heterogeneous structural conditions of the rehabilitation facilities is perceived and realized. In light of the requirement for biopsychosocial therapy concepts, there is therefore a need for more in-depth analyses of facilitating factors and barriers in order to identify optimum teamwork opportunities for the large number of different conditions that prevail in rehabilitation facilities. As a well-functioning team is an essential condition for achieving exercise therapy goals as well, it is necessary to evaluate the exercise therapy teamwork in order to reflect its importance for intervention outcomes and to derive practical implications for achieving effective physical activity promotion.

The goal of this paper is to gain an insight into the status quo of teamwork processes in rehabilitation practice in Germany. Based on a nationwide survey of exercise therapy in rehabilitation programs using a mixed methods design, this paper is thus dedicated to answer the following research questions in an explorative way.

Based on the quantitative part of the study: How often are interprofessional and exercise therapy team meetings held in rehabilitation facilities, and what topics are covered? Do the other professions pass on information about the rehabilitants to the exercise therapists? How do exercise therapists perceive the quality of interprofessional team meetings? (See Results 3.1.)

Based on the qualitative part of the study: What are the facilitating factors and the barriers for teamwork? What are central team-related process features? (See Results 3.2.)

Based on a combination of the quantitative and qualitative part of the study: How are the aforementioned team-related process features and their facilitating factors and barriers linked to (a) structural features of the exercise therapy department (e.g., health condition, form of care, size of the department, educational background of the head) on the one hand (see Results 3.3) and (b) the perceived satisfaction with the interprofessional teamwork on the other hand (see Results 3.4)?

## 2. Methods

### 2.1. Overall Design of the Study

The project “Exercise therapy in medical rehabilitation: a survey at facility and practitioner level” was implemented using a sequential explanatory mixed method design [18]. In the two consecutive parts of the study, quantitative and qualitative methods were integrated. A detailed description of the study protocol can be found in Geidl et al. [19].

The first part of the study used a standardized, quantitative written survey of the heads of exercise therapy departments to compile a comprehensive national overview of conceptual and process-related features of exercise therapy at the level of individual rehabilitation facilities. Based on this questionnaire-based cross-sectional survey, the second part of the study involved recording the subjective views of exercise therapy practitioners in a more differentiated manner. For this purpose, two one-and-a-half day workshops were held. Fifty-eight exercise therapy practitioners from different facilities held discussions on various topics related to physical activity promotion in exercise therapy in six disease-specific focus groups. Based on Kuckartz [20], the quantitative and qualitative parts of the study were first analyzed separately before combining the findings for both parts.

There is a positive ethics vote by the independent Ethics Commission of the Medical Faculty of Friedrich-Alexander University Erlangen-Nuremberg (Invoice no. 182_16B) for the study. The study was carried out in accordance with the Declaration of Helsinki and the Guideline for Good Clinical Practice (e.g., informed consent, voluntary nature, data protection). The participants provided a written declaration of consent before participating in the study.

### 2.2. Quantitative Part of the Study

#### 2.2.1. Design and Implementation

For the questionnaire-based cross-sectional survey, heads from a total of 1558 exercise therapy departments in 1146 rehabilitation facilities were contacted in writing via the German Statutory Pension Insurance (Deutsche Rentenversicherung Bund, DRV Bund) in May 2015. All in-patient, day-care, and out-patient facilities for all health conditions that took part in the quality assurance process of DRV Bund were contacted in writing. The questionnaire was returned directly to the leading scientific research institute in pseudonymized form.

#### 2.2.2. Return and Sampling

Overall, 541 of the 1146 rehabilitation facilities (47.2%) provided information on their exercise therapy departments (734 questionnaires). Of the 734 questionnaires, 21 had to be excluded, as they were not completed properly, leaving 713 questionnaires for the subsequent analyses.

Table 1 provides an overview of the exercise therapy departments and heads taking part in the survey. The vast majority of the questionnaires stemmed from in-patient and day-care facilities (71%). The size of the department was operationalized based on the number of rehabilitation patients to be treated per week. The classification into smaller or larger departments was made using the median split (i.e., departments that treated less than 130 patients per week in average were considered as smaller). The largest proportion of the questionnaires (i.e., 44.2%) involved orthopedic diseases.

#### 2.2.3. Data Source and Data Analysis

Structural features of the exercise therapy departments and team-related process features were measured based on the recording of structural and process features in out-patient neurorehabilitation [21].

For the following evaluations, the *structural features of exercise therapy departments* were classified dichotomously:

- Health conditions: more somatic (e.g., orthopedics, cardiology, neurology) vs. more psychological (psychosomatic, addiction)
- Form of care: (day-care or) in-patient vs. out-patient
- Department size: smaller vs. larger department
- Educational background of the head: physiotherapy vs. exercise therapy/sports science.

Team-related process features:

Exercise therapy heads stated the *frequency of exercise therapy team meetings* and *interprofessional meetings* in the rehabilitation team as a whole using a closed response format (at least once a week, every two weeks, approx. once a month). To improve the ability to analyze and interpret the data, another dichotomous classification was made (weekly vs. not weekly). In addition, respondents were asked to grade the *frequency of topics of exercise therapy team meetings* (case discussions and agreeing on the individual therapy process, information/exchange of the results of tests/measurements used, refinement of the exercise therapy concept, organizational points) using a four-level scale. Here too, the data were dichotomized (very frequent/frequent vs. sometimes/seldom). The same procedure was chosen for assessing *setting exercise therapy goals in an interprofessional exchange* together in the rehabilitation team. In addition, respondents were asked in a dichotomous question whether *information* about the rehabilitation patients *is received as standard from other professional groups*.

*Interprofessional teamwork* was measured via the “Internal Participation Scale” (IPS) [22]. This short questionnaire comprises items from the areas of communication, coordination, cooperation, respect, and environment. The six items in total were graded on a four-level Likert scale (1 = Completely disagree, 2 = Disagree, 3 = Agree, 4 = Completely agree). There was also an option to tick “Don’t know”.

An overall score was calculated in each case using mean values, where one missing item was accepted. If several items were missing, no overall score was calculated. The scale had an acceptable internal consistency level (Cronbachs α = 0.85).

For the analyses, perceived quality of interprofessional teamwork was split into three categories for the distribution of the overall score (very positive valuation: IPS score = 4, positive valuation: 3.2 ≤ IPS score < 4, average valuation: IPS score < 3.2).

The data were evaluated using the program package SPSS (Version 22). The evaluations were based on those departments that had at least two full-time equivalents in total (n = 596). In addition to descriptive parameters, χ^2^ tests, and contingency coefficient C (of 0.10 ≤ weak, 0.30 ≤ average, 0.50 ≤ strong link) were calculated for correlation analyses.

### 2.3. Qualitative Part of the Study

#### 2.3.1. Design and Implementation

We applied focus group discussions, as these are suitable for capturing complex attitudes in multi-layered subject areas. A detailed description of the qualitative part of the study can also be found at Geidl et al. [23]. As part of the two workshops, semi-structured focus groups on various exercise therapy topics with a focus on physical activity promotion took place in April 2016 with exercise therapy practitioners for the health conditions Orthopedics back, Orthopedics total hip/knee replacement, Neurology, Oncology, Psychosomatics, and Addiction. The workshops were conducted by three scientific project workers. Because of the closeness of the facilitators to the research project, major emphasis was placed in advance on training and a pre-test in order to ensure the facilitators were open to the emergence of new topics and to practice their handling of extreme cases and unexpected situations.

#### 2.3.2. Sample

Exercise therapy heads who had already participated in the previously held nationwide, questionnaire-based, cross-sectional survey were contacted by post via DRV Bund using a coding list. In a two-phase invitation process, a total of 166 invitations were sent out, resulting in 73 registrations (return rate of 44%). The number of participants was limited to a total of 30 persons per workshop (each with three disease-specific groups with the group size being around 10 persons). This meant that a total of 13 persons had to be turned down.

A total of 58 persons (24 women, 34 men) aged between 28 and 61 (M = 45; SD = 10) took part. A detailed overview of the characteristics of the participants of the qualitative part of the study can be found at Geidl et al. [23].

#### 2.3.3. Data Source and Data Analysis

The data basis comprised six disease-specific focus groups on the topic of development trends in rehabilitation, including three themes: interdisciplinarity, patient gearing, and standardization/manualization. The significance and implementation of these themes were discussed (see also [19]). Within this paper, the focus lies on the results related to the topic of interdisciplinarity. Based on the sample described and a total scope of roughly eight hours and 30 min of discussion (on average 85 min per disease-specific group), data saturation can be assumed.

The focus groups were processed and interpreted using the structuring qualitative content analysis described by Kuckartz [20]. At the beginning, a word-for-word transcription of all focus group interviews took place based on the audio recordings using F4/5 software and in line with previously defined transcription rules. The content of the transcripts was encoded using the software MAXQDA (Version 12). To do this, main categories were formed a priori based on the lead questions after initiating text work. The text material was allocated to these main categories by two independent persons in accordance with the encoding rules set. To assess the category system, a preliminary consistency check was carried out, resulting in a revision of the category system involving a discussion of inconsistencies and differentiation of encoding rules. The sub-categories were determined inductively, and all of the text material was allocated by one person. To assess the sub-category system, 30% of the text material (two disease-specific groups) was encoded by a second independent person, and an intercoder agreement using the intercoder coefficient kappa was used to calculate 90% agreement of the segments. The kappa value for the sub-categories of interdisciplinarity stood at a good value of 0.64 for “Addiction” and a very good value of 0.87 for “Orthopedics total hip/knee replacement” [24], so there was no need for further revision of the category system. Based on systematically prepared thematic summaries to condense or reduce the material, the last step involved holding iterative discussions in the researcher team and developing core topics. For the later presentation of the results in English, the relevant text passages were translated by a native speaker who was fluent in both English and German.

## 3. Results

### 3.1. Descriptive Analyses

Based on the quantitative analyses, it can be said from a descriptive perspective that regular team meetings take place every week both within the exercise therapy department and at an interprofessional level in the rehabilitation team as a whole in 83.4% and 85.9% of the facilities (see Table 2).

The most frequent topic of exercise therapy team meetings was organizational points (84.6%). Other common topics were case discussions and agreeing on the individual therapy process (70.6%) and refinement of the exercise therapy concept (55%). Overall, the exchange about test results took place less frequently within these internal team meetings. Almost two thirds of the practitioners in exercise therapy departments (65.4%) reported that they received information about the rehabilitation patients beyond the diagnosis as standard from other professional groups at the beginning of the rehabilitation. In almost 60% of the departments, exercise therapy goals were set in an interprofessional exchange and together in the rehabilitation team as a whole.

Overall, exercise therapy heads rated the interprofessional teamwork very positively. Of the respondents, 30% gave the top score, while a further almost 50% rated the interprofessional teamwork as positive.

Based on the qualitative part of the study, a total of 695 text passages were encoded for the focus groups, of which 291 text passages related to the topic of interdisciplinarity. The core topics of the discussions were as follows:

- Team-related process features relating to the areas of communication and cooperation, as well as facilitating factors and barriers:
  - Interprofessional team meetings
  - Way of communication, interprofessional exchange and information flow
  - Exercise therapy team meetings and case discussions
- Valuation of teamwork

The results of the quantitative and qualitative part of the study are presented below, corresponding to these core topics. Table 3 gives an overview of team-related process features and their facilitating factors and barriers, as well as their links to structural features of the department and perceived quality of interprofessional teamwork.

### 3.2. In-Depth Understanding of Team-Related Process Features and Their Facilitating Factors and Barriers as Well as Their Links to Structural Features of the Department

#### 3.2.1. Interprofessional Team Meetings

Correlation analyses of the quantitative data showed a minor link between the frequency of interprofessional team meetings and the form of care (C = 0.11; *p* < 0.05). These meetings took place more frequently in in-patient rehabilitation facilities (see Table 4).

For interprofessional team meetings, the qualitative data showed that generally just one representative from each profession took part, as one participant said:

“You can’t bring everyone who works in the team to this team meeting. If you did, there would be no work done for an hour […]. That’s not ideal, but there is just no other way of doing it.” (#00:48:44-1# Orthopedics total hip/knee replacement)

This principle of sending representatives to the meetings was criticized in some cases and its effectiveness was questioned, as it appears to go hand-in-hand with a lack of communication within the professional groups, and the information flow and the passing on of information is often insufficient:

“Here, the team leads [physiotherapy etc.] meet once a week for an hour to discuss everything. […] That is passed on in the [internal, intraprofessional] team meetings. […] If I say something now, ten people leave here and everyone passes on a different account of what I said.” (#00:22:05-0# Orthopedics back)

In addition, these interprofessional team meetings reflected a hierarchical relationship, as often only heads took part. This was interpreted as a lack of appreciation for the other therapy practitioners.

It was emphasized that it is not the quantity of meetings, but the quality of the content that is decisive, and there appear to be shortcomings in this regard:

“So, as required, these weekly interdisciplinarity meetings are also implemented here. […] the framework exists, but in terms of content what happens here is rather questionable. […] And what we are working on now, to first of all bring back a little quality perhaps.” (#00:24:12-6# Neurology)

Good chairing of the meetings was seen as a facilitating factor to improve quality. Comments were often made that holding meetings might result in a reduction of therapy offerings due to a lack of resources (e.g., lack of time or personnel):

“Yes, but you have to consider what happens if you send 15 therapists into a meeting that lasts an hour. […] And depending on the size of the clinic, you simply have really tight capacities and with vacation, illness and everything else that goes along with that, it is not possible to always manage that. […] So many appointments are not carried out because of it.” (#01:04:14-7# Oncology)

There was broad consensus overall regarding the importance of team meetings, because they make a key contribution to improving the quality of care. However, there were critical comments that the right measure needed to be found in order to have enough time for active therapy. One possible proposed solution in the trade-off between time for therapy versus time for team meetings could be that meetings are held during the rehabilitation patients’ mealtimes:

“So we meet relatively often and I have to say it doesn’t really take away from patient time, at the cost of the patient. We really always only hold this team meeting when the patients are at breakfast or lunch.” (#00:53:18-7# Addiction)

#### 3.2.2. Way of Communication, Interprofessional Exchange, and Information Flow

Based on the quantitative data from the first part of the study, weak links were shown to exist (*p* < 0.05 in each case) between the interprofessional exchange of information and the form of care (C = 0.17), department size (C = 0.20), and health condition (C = 0.09). The exchange of information about the rehabilitation patients as standard at the beginning of the rehabilitation is associated with out-patient facilities, smaller departments, and health conditions of a more psychological nature.

The second part of the study also showed that interprofessional exchange appears to be a more predominant feature for health conditions of a more psychological nature:

“I also think it is very good, the exchange [with all professions] and also it gives me the chance to give my impressions from time to time or to get a bit of different information up front also about the patients. That is maybe also helpful to sometimes gauge behavior better.” (#00:53:51-0# Addiction)

In this way, it was possible to contribute own experiences and exchange pre-existing knowledge and to incorporate insights from other areas of therapy. Due to the fact that individual rehabilitation patients with health conditions of a more psychological nature displayed heterogeneous behavior patterns depending on the form of therapy, a need for more coordination was perceived, which one participant described as follows:

“The exchange is very intensive, and everyone is there. […] And many patients behave entirely differently in psychotherapy than in exercise therapy. Lots of nuances come to light there that the colleague from psychotherapy doesn’t get to see. And we always respect that and also consciously ask about it.” (#00:47:42-5# Addiction)

Interprofessional exchange was considered valuable, as it leads to information saturation and various aspects from different professions are taken into account. The therapy practitioners came to the conclusion that interprofessional knowledge or a knowledge of other therapy offerings foster this exchange. Another benefit of the exchange was seen as a reduction in incorrectly prescribed therapies. There was some discussion regarding how much decision-making scope the individual exercise practitioners have in prescribing therapy offerings and whether they have an opportunity to be part of the decision. In most facilities, the head doctor was perceived to be clearly dominant and the hierarchical stance of the head doctor in prescribing therapy was criticized.

For example, one-sided contact within a team was mentioned as a barrier for interprofessional exchange:

“It is simply the communication channels, if they are one-directional, it doesn’t work.” (#00:22:05-0# Orthopedics back)

This one-sided contact was repeatedly perceived negatively, and there was a desire for a mutual and equal exchange. The exchange with the medical staff was often criticized in this regard. That exchange appears to be more hierarchical with exercise therapists passing information to the medical staff, but rarely receiving information from them.

The size of the facility was also listed as a barrier for interprofessional exchange. A large number of rehabilitation patients leads, among other things, to a situation where not every therapy practitioner knows each individual patient personally and the exchange of information is thus more difficult, as one participant commented:

“As the therapist, I naturally do not know every patient in this large facility. And sometimes I think that’s a huge problem, […] because it is difficult to relay the information when I don’t really know them personally.” (#00:31:26-9# Orthopedics back)

On the whole, the attitude of management or the medical director to interprofessional teamwork appears to play a decisive role. It is more difficult for the exercise therapists to implement interprofessional teamwork if this is not supported by the clinic’s senior staff.

“I think the interdisciplinarity and importance given to it in a facility or a team always depends very heavily on the management of that facility.” (#00:54:31-9# Oncology)

#### 3.2.3. Exercise Therapy Team Meetings and Case Discussions

In terms of the frequency of exercise therapy team meetings, the analysis of quantitative data did not show any link to the structural features of the department. By contrast, links were found to exist for specific topics of exercise therapy team meetings (*p* < 0.05 in each case). For example, an informative exchange of the results of tests and measurement methods used took place more often for somatic diseases (C = 0.18), in smaller departments (C = 0.11), and in departments managed by physiotherapists (C = 0.18).

Organizational topics were mentioned more frequently in in-patient facilities (C = 0.12). The organizational efforts appear to be greater there, as one participant from an in-patient facility explained in the qualitative part of the study. Examples were listed of the organizational matters that have to be discussed.

“We have a separate slot for internal departmental [meetings], i.e., for issues like vacation applications to administration […] and that is where we discuss organizational matters. Defective devices, other matters, procurement of medical aids, broken exercise apparatus or other things.” (#00:31:06-5# Psychosomatic)

The quantitative analyses showed that case discussions were more frequent in out-patient (C = 0.13) and smaller departments (C = 0.16). Several of the exercise therapy practitioners from all health conditions raised the issue in the second part of the study of the size of the department and the number of rehabilitation patients requiring care as a decisive limiting factor for case meetings. They posed the question of a practicable department size in order to be able to hold case discussions for each patient:

“The aim of discussing all rehabilitation patients, I think that is utopian. That is not possible at all for us. And now I am wondering whether we shouldn’t be talking about a group size, or a facility size, where this would be even possible.” (#00:35:50-5# Orthopedics back)

Thus, owing to a lack of time, personnel, and space, often only problematic cases were discussed in the meetings:

“When a team meeting takes place, […] then really only the problematic patients are discussed, because it is not possible to discuss all ninety or one hundred patients in an hour.” (#00:48:44-1# Orthopedics total hip/knee replacement)

These case discussions resembled a mere passing on of information rather than an involved discussion, which was mainly due to time pressure. As an idea to overcome this problem, a suggestion was made to brief staff on planned case discussions beforehand to allow information to be gathered in advance in a targeted way.

### 3.3. Links between Team-Related Process Features and Structural Featrues of the Exercise Therapy Department

Table 4 displays associations between the team-related process features and their facilitating factors and barriers and structural features of the exercise therapy department (e.g., health condition, form of care, size of the department, educational background of the head).

### 3.4. Links between Team-Related Process Features and Perceived Quality of Interprofessional Teamwork

Based on the quantitative data, links were analyzed that showed that an exchange of information about the rehabilitation patients as standard is associated with a very positive valuation of interprofessional teamwork (C = 0.11) (Table 5) and of interprofessional setting of therapy goals (C = 0.24). Further links were found to exist between specific topics in exercise therapy team meetings and perceived teamwork. Case discussions (C = 0.24), an informative exchange of the results of tests and measurement methods (C = 0.27), and the refinement of exercise therapy concepts (C = 0.13) were linked to a more positive valuation of teamwork. By contrast, the analysis of the quantitative data showed that the frequency of both exercise therapy team meetings and intraprofessional team meetings in the rehabilitation team as a whole is not linked to the perceived teamwork.

Qualitative data confirm a positive attitude towards interprofessional teamwork but also a desire for more recognition, as expressed by one participant:

“But I think we simply have to have […] more scope for meetings. And that has to be recognized by the cost payer, otherwise it won’t happen […] and there is no longer any scope for an exchange. And I think that is really the advantage of our in-patient work. Many disciplines, but not in the sense of too many cooks, but many disciplines achieve a super result when they work together.” (#00:17:29-8# Psychosomatic)

## 4. Discussion

Exercise therapy teamwork and a well-functioning team is an essential basis for achieving goals of multimodal rehabilitation programs, like the promotion of rehabilitants’ physical activity. The nationwide survey allows for a comprehensive insight into the status quo of teamwork in exercise therapy, with roughly half of the rehabilitation facilities in Germany included in the survey.

Heads of the exercise therapy departments of these facilities assessed interprofessional teamwork as good to very good on the whole, both in the quantitative cross-sectional survey and in the qualitative second part of the study. This corresponds to the findings of other reviews in which differences were found to exist in the valuation of interprofessional teamwork in different professional groups. Exercise therapists assessed teamwork higher compared to other professional groups, such as nursing care staff [25,26]. Compared to studies by Körner and colleagues [26], the interprofessional teamwork was exceeded on average in this national survey. Although it is possible that the team leader role of those surveyed may have influenced the assessment in terms of a positive self-assessment, it can be said that the heads in exercise therapy departments consider the interprofessional teamwork to be comparatively good. However, it is not possible to sufficiently assess the extent to which facilities did not take part in the survey for systematic reasons connected to teamwork, and to what extent a greater spread in the valuation of interprofessional teamwork can be expected in the entirety of the facilities. Since the information comes from the heads of exercise therapy departments, it is not clear whether practicing exercise therapists without a management function also share the positive assessment of teamwork.

Based on the quantitative data of the first part of the study, links between team-related process features and the perceived quality of interprofessional teamwork were analyzed. Before discussing these results in detail, it is important to note that this study did not include indicators of rehabilitation outcomes. A general limitation of the study is therefore that the actual relationship between team process features and patient outcomes cannot be analyzed. Bearing this is mind, the results showed that the interprofessional setting of therapy goals is associated with a very positive valuation of teamwork. Moreover, there is no doubt that regular team meetings are considered very important for teamwork and are well-established in the significant majority of the facilities surveyed. An equally clear and noteworthy finding was that there is no link between the frequency of exercise therapy meetings alone and the structural features and satisfaction with teamwork. The results of the focus groups affirm that the regular team meetings are seen as a central platform for exchange for coordinating teamwork. However, it appears that in a minority of the rehabilitation facilities it is not standard to have team meetings at least once a week. Yet the framework concept for medical rehabilitation of the most important cost providers for rehabilitation programs in Germany [3] considers this necessary for the implementation of a holistic rehabilitation approach with the central goal of physical activity promotion in interprofessional teams. The results of the focus groups allow for some reasons to be identified for the deviations from the regulations in the framework concept. For example, there was broad consensus on the importance of team meetings in terms of improving the quality of care. Meanwhile, there was a critical discussion of the fact that a lack of time, personnel, and space meant that having meetings often resulted in a reduction of therapy offerings, or that only problematic cases were discussed in meetings as a result. Meeting times and therapy times are perceived to be in clear conflict with each other. By analogy, earlier studies showed that insufficient time slots for liaising as part of a team were seen as preventing interprofessional team work [12,13,27]. 

Interprofessional meetings were often carried out on a representative basis. This process was criticized in the second part of the study, as it was often linked to a loss of information. Furthermore, therapy practitioners without an executive function might get the impression of not being recognized within the team. Against this backdrop, the recommendation from the framework concept of DRV Bund that all team members should have a right to speak is desirable, but may have been worded too idealistically, and would appear to contradict what happens in daily rehabilitation practice. Ensuring a sufficient flow of information while using the practice of sending representatives to meetings poses an additional challenge here.

In terms of the exercise therapy team meetings in relation to their content aspects, the findings from the first part of the study showed a link between the topics and structural features of the rehabilitation facilities. In view of this fact, it is significant that case discussions take place more frequently in out-patient facilities and smaller departments. Confirmed by the focus group discussions, a couple of aspects were highlighted—for instance, in large departments, insufficient resources meant that it was not possible to discuss all patients individually. Instead, only problematic cases were discussed. To remedy this problem, the qualitative analysis of the focus groups suggest to brief staff in advance on which patients would be discussed so that information could be gathered beforehand in a targeted manner. This is also in line with the findings of Verhaegh et al. [28], who see the preparation of case discussions as a key element to their effective implementation, so that all relevant information can be gathered in advance. So there is a discussion around whether, in general, criteria for relevant (problem) cases of a meeting should be set out internally that leave scope for positive developments of rehabilitation patients.

Further links in terms of the exercise therapy team meetings were found as well between specific topics and the perceived quality of interprofessional teamwork. Thus, the refinement of exercise therapy concepts was linked to a more positive evaluation of teamwork. However, the dissemination of evidence-based concepts for physical activity promotion has reached only half of the exercise therapy departments [29], among other things, because it is difficult to discuss and plan those concepts in team meetings.

In terms of the implementation of team meetings, it can be said in general that there appear to be success features for their effectiveness. For example, it was shown in the qualitative part that good chairing of the meetings was seen as a facilitating factor. In addition to the chairing of the meeting, the literature (e.g., [30]) contains further factors for success, such as clear meeting objectives, respectful discussions, and multilateral communication of all team members, mutual decision-making (instead of a mere exchange of information), use of a common language or minutes of the meeting.

A finding from the quantitative analyses showed that the passing on of information from other professional groups, which constitutes a team-based procedural determinant of effective teamwork, is associated with an out-patient setting, smaller departments, and health conditions of a psychological nature. In this context, therapy practitioners having an interprofessional knowledge or a knowledge of other therapy offerings was seen as a facilitating factor for exchange. This is in line with findings from other studies (e.g., [14,27,31]). A barrier of interprofessional exchange cited in the second part of the study was a one-sided contact within the team, and there was a desire for a mutual and equal exchange. In particular, communication with medical practitioners appears to be informed more by hierarchy and by therapy practitioners doing the groundwork. Because different professions generally have different styles of communication, this can lead to communication problems per se [32], because people are not speaking the same “language” [27]. International studies show that professional groups often tend to stay within their own “silo”. Accordingly, their method of working is much more intraprofessional than exchanging information with other professions [25,33], as this ensures a commonality of language, procedures, and attitudes. There is a recognition of a need to generally structure training content for the specialist health professions in an interprofessional manner (“interprofessional education”) in order to lay the foundations for later cooperation and to encourage professional groups to look outside their own narrow professional “silo” [34].

## 5. Conclusions

Our results contribute to a deeper understanding of teamwork in exercise therapy as a basis for successful implementation of biopsychosocial therapy concepts within multimodal rehabilitation programs. The chosen methodological approach in the mixed methods design integrated quantitative and qualitative data, thereby enabling a multilayered insight into the status quo of teamwork processes in Germany. In this way, it was possible to identify facilitators and barriers for team-related processes in exercise therapy and their links to structural features of the exercise therapy departments and the perceived quality of interprofessional teamwork. These detailed findings form the essential basis for the systematic development of quality exercise therapy in rehabilitation.

Structural and organizational conditions (such as the department size or sufficient time slots for liaising on content) had an impact on team-based processes. Consequently, adequate framework conditions are essential for effective teamwork. Thereby, the different structural features of the exercise therapy departments should always be taken into account to optimize teamwork. For further development, framework concepts for team meetings could be developed, building on the insights of recent models of integrated team leadership [35]. A health system that facilitates and promotes interprofessional teamwork is a key requirement in this context [36]. However, further research should examine the links between perceived effective teamwork and actual rehabilitation outcomes achieved. Such studies would substantially expand the general body of knowledge about this relationship specific to the role of effective teamwork in exercise therapy. Thereby, it is of particular importance to elaborate the generalizability of the findings to other rehabilitation settings beyond Germany.

## Figures and Tables

**Table 1:** Characteristics of the responding exercise therapy departments of the quantitative part of the study (n = 713).

	Frequency	
Characteristic	Number	%
Form of care		
In-patient (and day-care)	506	71.0
Out-patient	175	24.5
Missing	32	4.5
Department size		
Smaller departments (<= 130 rehabilitation patients/weak)	349	48.9
Lager departments (> 130 rehabilitation patients/weak)	338	47.4
Missing	26	3.6
Educational background of the exercise therapy department heads		
Physiotherapy	300	42.1
Exercise therapy/sports science	283	39.7
Others	93	13.0
Missing	37	5.2
Disease-specification		
Orthopedics total	315	44.2
Orthopedics back	149	20.9
Orthopedics total hip/knee replacement	97	13.6
Orthopedics mixed	69	9.7
Addiction	119	16.7
Psychosomatic	83	11.6
Cardiology	60	8.4
Neurology	52	7.3
Oncology	45	6.3
Pneumology	13	1.8
Endocrinology	10	1.4
Missing	16	2.2
Health conditions		
More somatic (e.g., orthopedics, cardiology, neurology)	495	69.4
More psychological (psychosomatic, addiction)	202	28.3
Missing	16	2.2

**Table 2:** Descriptive parameters of team-related process features of the quantitative part of the study for departments that had at least two full-time equivalents in total (n = 596).

	Frequency	
Team-Related Process Feature	Number	%
Interprofessional meetings in the rehabilitation team as a whole		
Once a week	512	85.9
Less than once a week	79	13.3
Missing	5	0.8
Exercise therapy team meetings		
Once a week	497	83.4
Less than once a week	93	15.6
Missing	6	1.0
Topics of exercise team meetings		
Case discussions and agreeing on the individual therapy process		
(Very) frequent	421	70.6
Sometimes/seldom	155	26.0
Missing	20	3.4
Information/exchange of the results and the tests/assessments results used		
(Very) frequent	228	38.3
Sometimes/seldom	324	54.4
Missing	44	7.4
Refinement of the exercise therapy concept/offer		
(Very) frequent	328	55.0
Sometimes/seldom	243	40.8
Missing	25	4.2
Organizational aspects		
(Very) frequent	504	84.6
Sometimes/seldom	64	10.7
Missing	28	4.7
Setting exercise therapy goals in an interprofessional exchange		
(Very) frequent	354	59.4
Sometimes/seldom	212	35.6
Missing	30	5.0
Information about the rehabilitation patients from other professional groups is received as		
Standard	390	65.4
Not standard	200	33.6
Missing	6	1.0
Interprofessional teamwork (IPS-Score: M = 3.59; SD = 0.42)		
Very positive valuation (IPS-Score = 4)	179	30.0
Positive valuation (3.2 ≥ IPS-Score < 4)	294	49.3
Average valuation (IPS-Score < 3.2)	118	19.8
Missing	5	0.8

Note. IPS = Internal Participation Scale.

**Table 3:** Overview of team-related process features and their facilitating factors and barriers, as well as their links to structural features of the department and to perceived quality of interprofessional teamwork.

Team-Related Process Feature	Facilitating Factors	Barriers	Link to Structural Features of the Department (see also Table 5)	Link to Perceived Interprofessional Teamwork (see also Table 5)
Interprofessional team meetings	- Quality of the content before quantity of meetings - Good chairing	- Lack of time - Lack of personnel - Sending just one representative from each profession results in -> Lack of communication -> Insufficient information flow and passing on of information -> Hierarchy -> Lack of appreciation	Interprofessional team meetings were more frequently in in-patient facilities (weak association, see also Table 5)	No associations found
Way of communication, interprofessional exchange and information flow	- Interprofessional knowledge/knowledge for other therapy offerings - Mutual and equal exchange - Willingness at management level	- Hierarchy - One-sided contact - (only in the direction of the medical staff) - Large size of facility - Knowing patients not personally	Interprofessional exchange of information as standard was associated with - Out-patient facilities (weak association) - Smaller departments (weak association) - Health conditions of a more psychological nature (weak association)	Interprofessional exchange of information as standard was associated with a very positive valuation of teamwork (weak association)
Exercise therapy meetings and case discussions	- Brief staff on planned case discussions - Obtaining information in advance	- Lack of time - Lack of personnel - Lack of space - Large size of department - Large number of patients	No links found for exercise therapy meetings (but for specific topics) Case discussions were more frequent in out-patient and smaller departments (weak associations)	No associations found for exercise therapy meetings (but for specific topics) Case discussions, information/exchange of results of tests and refinement of concepts were associated to a more positive valuation of teamwork (weak associations)

**Table 4:** χ^2^ tests and contingency coefficient C for correlation analyses between team-related process features and structural features of exercise therapy departments.

Team Related Process Features	Health Conditions				Form of Care				Department Size				Educational Background of the Head of Department			
	More Somatic	More Psychological			In-Patient	Out- Patient			Large	Small			Physio-Therapy	Exercise Therapy/ Sports Science		
			*p*	C			*p*	C			*p*	C			*p*	C
Exercise therapy team meetings (n)	469	107			415	154			333	236			277	219		
Once a week (in %)	84.9	84.1	0.85	0.01	85.8	79.2	0.06	0.08	85.9	81.8	0.19	0.06	87.7	81.3	0.06	0.09
Topics exercise team meetings																
Case discussions (n)	458	103			407	149			326	229			273	210		
(Very) frequent (in %)	**75.3**	66.0	0.05	0.08	69.0	**82.6**	0.00	0.13	66.9	**81.2**	0.00	0.16	75.5	70.0	0.18	0.06
Results of assessments (n)	444	94			390	144			314	218			263	200		
(Very) frequent (in %)	**45.7**	22.3	0.00	0.18	39.0	45.1	0.20	0.06	36.6	**47.7**	0.01	0.11	**48.3**	30.5	0.00	0.18
Refinement of concepts (n)	453	103			404	147			325	225			269	210		
(Very) frequent (in %)	57.4	54.4	0.58	0.02	55.7	61.9	0.19	0.06	56.9	58.2	0.76	0.01	55.8	59.1	0.47	0.03
Organizational points (n)	452	104			402	148			320	229			270	209		
(Very) frequent (in %)	88.5	88.5	0.99	0.00	**91.0**	82.4	0.01	0.12	90.6	85.6	0.07	0.08	87.4	91.4	0.17	0.06
Interprofessional meetings in the rehabilitation team as a whole (n)	468	108			415	155			331	239			277	220		
Once a week (in %)	85.9	89.8	0.28	0.05	**88.7**	80.0	0.01	0.11	88.2	84.9	0.25	0.05	87.0	88.6	0.58	0.03
Setting goals in an interprofessional exchange (n)	447	106			400	148			317	229			262	212		
(Very) frequent (in %)	63.1	60.4	0.60	0.02	58.5	**73.0**	0.00	0.13	54.3	**72.9**	0.00	0.19	65.7	57.1	0.06	0.09
Information from other professional groups is received as (n)	468	107			416	153			332	236			277	219		
Standard (in %)	64.1	**74.8**	0.04	0.09	61.1	**79.1**	0.00	0.17	58.7	**78.0**	0.00	0.20	63.2	69.4	0.15	0.07

Note. n = Number; C = contingency coefficient. in bold: direction of significant correlations (*p* < 0.05).

**Table 5:** Calculated χ^2^ tests and contingency coefficient C for correlation analyses between team-related process features and the perceived quality of interprofessional teamwork.

Team-Related Process Features	Interprofessional Teamwork (via the IPS-Score)				
	Average Valuation	Positive Valuation	Very Positive Valuation		
				*p*	C
Exercise therapy team meetings (n)	116	291	179		
Once a week (in %)	86.2	83.5	84.9	0.78	0.03
Topics of exercise team meetings					
Case discussions (n)	114	274	174		
(Very) frequent (in %)	55.3	75.2	**86.2**	0.00	0.24
Results of tests/assessments (n)	109	274	165		
(Very) frequent (in %)	20.2	39.4	**59.4**	0.00	0.27
Refinement of concepts (n)	113	280	174		
(Very) frequent (in %)	46.9	57.1	**65.5**	0.01	0.13
Organizational points (n)	116	276	172		
(Very) frequent (in %)	88.8	87.0	91.3	0.37	0.06
Interprofessional meetings in the rehabilitation team as a whole (n)	116	292	179		
Once a week (in %)	91.4	87.3	82.7	0.09	0.09
Setting goals in an interprofessional exchange (n)	114	277	171		
(Very) frequent (in %)	43.0	74.9	**77.2**	0.00	0.24
Information from other professional groups is received as (n)	118	289	178		
Standard (in %)	58.5	65.1	**73.0**	0.03	0.11

Note. C = contingency coefficient; IPS = Internal Participation Scale. in bold: direction of significant correlations (*p* < 0.05); n = Number.

## Data Availability

The data generated during Phase 1 are available from the corresponding author on reasonable request. Interview data of Phase 2 may be linked to individuals interviewed and as such is not available open use.
